# Liver cirrhosis in HIV/HCV‐coinfected individuals is related to NK cell dysfunction and exhaustion, but not to an impaired NK cell modulation by CD4^+^ T‐cells

**DOI:** 10.1002/jia2.25375

**Published:** 2019-09-19

**Authors:** María L Polo, Yanina A Ghiglione, Jimena P Salido, Alejandra Urioste, Gabriela Poblete, Alicia E Sisto, Ana Martinez, María J Rolón, Diego S Ojeda, Pedro E Cahn, Gabriela J Turk, Natalia L Laufer

**Affiliations:** ^1^ CONICET‐Universidad de Buenos Aires Instituto de Investigaciones Biomédicas en Retrovirus y Sida (INBIRS) Buenos Aires Argentina; ^2^ Infectious Diseases Unit Hospital General de Agudos “Dr. JA Fernández” Buenos Aires Argentina; ^3^ Gastroenterology Unit Hospital General de Agudos “Dr. JA Fernández” Buenos Aires Argentina; ^4^ Fundación Huésped Buenos Aires Argentina

**Keywords:** HIV, hepatitis C virus, liver fibrosis, NK cells, CD4‐positive T‐lymphocytes, Immunology

## Abstract

**Introduction:**

HIV worsens HCV‐related liver disease by accelerating fibrosis progression; however, progression rates are extremely variable among HIV/HCV‐coinfected individuals. NK cells are associated with modulation of liver fibrosis and are profoundly altered during HCV and HIV infections. CD4^+^ T‐cells modulate NK cell function, and are also affected by HIV infection. Here, we aim to characterize the association of hepatic fibrosis with both the phenotype and function of peripheral NK cells and their regulation by CD4^+^ T‐cells, in HIV/HCV‐coinfected individuals.

**Methods:**

Thirty‐four HIV/HCV‐coinfected individuals with minimal (n = 16) and advanced (n = 18) fibrosis (METAVIR F0/F1 and F4 scores respectively) and 20 healthy volunteers were enrolled. PBMC were obtained from peripheral blood samples and NK and CD4^+^ T‐cells were isolated and analysed. NK cell phenotype (CD25, CD69, Nkp46, NKG2D, PD‐1), degranulation (CD107a) and IFN‐γ and TNF‐α production, as well as CD4^+^ T‐cell activation (CD69, CD25 and CD38) were measured by flow cytometry. CD4^+^ T‐cell conditioned medium (CM) derived from F0/F1 or F4 individuals was assessed for IL‐2 levels by ELISA. Modulation of NK cell functionality by these CMs was also analysed.

**Results:**

When comparing to NK cells from individuals with minimal fibrosis, degranulation and cytokine secretion by NK cells from subjects with F4 scores was significantly impaired, while PD‐1 expression was augmented. On the one hand, neither the expression of activation markers nor IL‐2 secretion was distinctly induced in CD4^+^ T‐cells from subjects with F0/F1 or F4 METAVIR scores. Finally, NK cell degranulation and cytokine secretion were not differentially modulated by CD4^+^ T‐cell CM, whether CD4^+^ T‐cells derived from subjects with minimal or advanced fibrosis.

**Conclusions:**

Low levels of NK and CD4^+^ T‐cells in HIV/HCV‐coinfected individuals with advanced liver fibrosis have been previously described. Here, we show that advanced liver fibrosis in coinfected individuals is associated to a defective function of NK cells and an increased expression of the exhaustion/senescence marker PD‐1. This NK signature could not be attributed to changes in the ability of CD4^+^ T‐cells to modulate NK cell function.

## 1 Introduction

Nearly 71 million people worldwide are chronically infected with the hepatitis C virus (HCV) 1. Owing to overlapping modes of transmission, coinfection with HCV and HIV is frequent. Globally, 2.3 million individuals are estimated to be coinfected with both viruses, with prevalence ranging between 10% and 90%, depending on populations and geographical regions analysed 2. Despite antiretroviral therapy (ART), HIV‐coinfected individuals present an accelerated course of HCV‐related liver disease compared to HCV‐monoinfected individuals 3, 4; immunosuppression and chronic inflammation are some of the causes of such aggravation 5. Additionally, among HIV/HCV‐coinfected individuals, some subjects progress to cirrhosis and others do not develop any degree of liver fibrosis in their life time. Factors involved in such heterogeneity remain unidentified.

In humans, natural killer (NK) cells represent approximately 10% of peripheral blood mononuclear cells, but up to 30% to 50% of intrahepatic lymphoid cells 6. As effectors of the innate immune system, NK cells target virally infected or transformed cells by mediating cytotoxicity and producing proinflammatory cytokines. Additionally, cumulative evidence has revealed that NK cells play an important role in inhibiting hepatic fibrosis 7. Due to chronic hepatic injury, hepatic stellate cells (HSCs) are activated, which leads to extracellular matrix deposition, fibrosis and eventually, cirrhosis. Independent studies in both preclinical and clinical models have reported that NK cells kill early activated or senescence‐activated HSCs by releasing cytotoxic granules or inducing death signals through the expression of death receptors 8, 9, 10, 11, 12, 13, 14. Secreted‐IFN‐γ also enhances antifibrotic effect of NK cells by inducing HSCs cycle arrest and apoptosis 15. Conversely, it has been suggested that HSCs are capable of impairing NK cell antifibrotic capacity 16, 17.

Chronic HCV and HIV infections leave a distinct signature on peripheral blood NK cells 18, 19, and so, alterations of NK cell phenotype and/or function were suggested as sensitive biomarkers of viral exposure. To date, whether progression of hepatic fibrosis in coinfected individuals is associated to a defective NK function has not been demonstrated. In a previous study, we described that HIV/HCV‐coinfected individuals with advanced fibrosis presented lower percentages of CD4^+^ T‐cells and NK cells compared with subjects with minimal fibrosis 20. Here, we show for the first time that progression of liver fibrosis is associated to a defective NK cell function that could not be attributed to a defective modulation by CD4^+^ T‐cells.

## 2 Methods

Healthy volunteers (HV, n = 20) and HIV/HCV‐coinfected individuals (n = 34) on follow‐up at a Public Hospital Infectious Diseases Unit, Buenos Aires, Argentina, were included in this study. Written Informed Consent was obtained. Blood samples were withdrawn at INBIRS Institute. The study was conducted in accordance with the Declaration of Helsinki, and was approved by the Bioethics Committee of Fundación Huésped. Sample collection and processing were performed from July 2016 to July 2018.

HIV/HCV‐coinfected individuals were classified based on their level of fibrosis according to transient hepatic elastography (FibroScan^®^ and SuperSonic Imagine's Aixplorer^®^). Subjects with a result of ≤7.1 Kpa were classified as compatible with a METAVIR score of F0/F1: absent or minimal fibrosis and those with ≥12.5 Kpa as compatible with METAVIR F4: cirrhosis 21, 22, 23. Individuals enrolled in this study were not acutely or chronically HBV‐infected (determined by serology); and denied current use of recreational drugs and more than 14 units/week of alcohol intake on a regular basis. HV were recruited among hospital employees. To be included they had to present a value of ≤5 Kpa on transient hepatic elastography, had negative HIV/HCV/HBV serology, no history of alcohol, illicit drug consumption and other clinically relevant conditions.

### 2.1 Isolation of PBMC, NK and CD4^+^ T‐cells

See Supporting Information.

### 2.2 Activation of CD4^+^ T‐cells

For CD4^+^ T‐cell activation, 100,000 freshly isolated CD4^+^ T‐cells were incubated with Dynabeads Human T‐Activator CD3/CD28 (Thermo Fisher, USA) at different bead‐to‐cell ratios, for different times. Then, cells were processed by flow cytometry to determine CD69, CD25 and CD38 expression. Conditioned medium from activated CD4^+^ T‐cells (CM) were collected, and stored at −80°C.

For NK cytokine secretion assays, CM from CD4^+^ T‐cells of a selected HV was used. Selection of healthy CD4^+^ T‐cells was performed after certifying that activation was efficiently induced by anti‐CD3/CD28 beads (Figure S1A). To obtain adequate amounts of material, supernatants from activated (bead‐to‐cell ratio of 1:1, 48 hours) CD4^+^ T‐cells of the selected donor were obtained on two different occasions, and subsequently pooled.

### 2.3 Cytokine secretion

See Supporting Information.

### 2.4 CD107a assay

For NK cell degranulation assays, the K562 cell line was used as a sensible target at an effector to target ratio of 1:1. PBMC were thawed and cultured overnight in cRPMI, at 37°C with 5% CO2. Next, one million viable PBMC were coincubated for five hours with K562 cells, in the presence of anti‐CD107a‐FITCmAb, brefeldin and monensin (4 μL, 10 μg/mL and 0.7 μg/mL respectively, BD Biosciences, San Diego, CA, USA). To assess the basal levels of degranulation, PBMC were incubated in the absence of K562 cells.

Afterwards, we evaluated the effect of CM from CD4^+^ T‐cells of HIV/HCV‐coinfected individuals on healthy NK cells. To avoid inter‐subject bias, NK cells purified from a single HV were used as effector. Selection of healthy NK cells was performed after certifying that degranulation capacity was efficiently induced by K562 cells (Figure S1B). Isolated NK cells were pretreated for 18 hours with CM from activated CD4^+^ T‐cells. Then, 100,000 viable NK cells were exposed to either K562 cells or cRPMI, as formerly described.

### 2.5 IFN‐γ and TNF‐α secretion

For cytokine secretion assays, either PBMC or purified NK cells were used. PBMC from HCV/HIV‐coinfected individuals were pretreated with either cRPMI or CM from activated CD4^+^ T‐cells from a selected HV. On the one hand, NK cells from a single volunteer were pretreated with CM from HIV/HCV‐coinfected individuals. After 18 hours, cells were coincubated with the K562 cell line, as described in the previous section.

### 2.6 Flow cytometric analysis

Cells were immunophenotyped by flow cytometry on a FACS Canto Flow Cytometer (BD Biosciences). For antibodies and gating strategies, see Table S1, Figure S2. Flow cytometry data were analysed by FlowJo software v.10 (FlowJo Enterprise, Treestar Inc., Ashland, OR, USA). Phenotype and functionality assays were performed according to cells availability.

### 2.7 Statistical analysis

Statistical analyses were performed using GraphPad Prism v.7.0 (GraphPad Software Inc., San Diego, CA, USA) and SPSS software v.19.0 (SPSS Corp., Armonk, NY, USA). Parametric and non‐parametric tests were used as appropriate, for details; see Supporting Information. Significance was assumed at *p *<* *0.05. (*) *p* < 0.05, (**) *p* < 0.01 and (***) *p* < 0.001.

## 3 Results

### 3.1 Frequency of NK and CD4^+^ T‐cells is severely diminished in HIV/HCV‐coinfected individuals with advanced fibrosis

This study enrolled HIV/HCV‐coinfected individuals with minimal or advanced fibrosis. All HIV‐coinfected individuals were on ART with undetectable HIV viral load, with no difference in the time on ART between groups. One third of the individuals in the advanced fibrosis group and 14% with minimal fibrosis received IFN‐based HCV treatment, with a median of eight years (IQR eight to eleven years) before sampling, and none of them achieve sustained virologic response. Age, time of known HIV or HCV infection, and HCV genotypes were similar between individuals (Table 1). As expected, F4 group was positively associated to liver stiffness, AST, γ‐GT, and total bilirubin levels; and negatively to platelet count, albumin levels and prothrombin time (Table 1). Male sex has been shown to be positively related with liver fibrosis progression in HCV‐monoinfected or HIV/HCV‐coinfected individuals 24, 25. Nevertheless, neither NK nor CD4^+^ T‐cell frequencies were associated with gender in our study.

**Table 1:** Subjects characteristics

Characteristics	Control, n = 20	F0/F1, n = 16	F4, n = 18	*p* value
Age (years)^a^	44.4 (26 to 70)	47.1 (23 to 57)	51.2 (35 to 64)	0.20
Female sex (%)^b^	9 (45)	7 (44)	4 (22)	0.12
Prior IDU (%)	‐	11 (69)	15 (82)	0.42
CD4 count (cells/μL)^a^	1126.0 (629 to 2037)^c^	716.6 (153 to 1412)	563.8 (54 to 1278)^c^	0.002
CD8 count (cells/μL)^a^	722.4 (348 to 1566)	877.1 (321 to 1812)	947.8 (253 to 2230)	0.73
Known Time HCV infection (years)^a^	‐	16.0 (1 to 25)	16.3 (3 to 31)	0.67
Known time HIV infection (years)^a^	‐	19.9 (9 to 31)	19.3 (3 to 30)	0.80
Time of ART (years)^a^	‐	12.4 (7 to 17)	13.6 (8 to 20)	0.60
Previous use of ddI/d4T	‐	3 (20)	4 (25)	>0.99
Present ART based on PI	‐	7 (46)	7 (38)	>0.99
Present ART based on NNRTI	‐	5 (31)	2 (12)	0.21
Present ART based on INSTI	‐	4 (23)	9 (50)	0.17
Previous IFN/PEGIFN	‐	3 (14)	6 (33)	0.44
HCV genotype (%)^a^				
Genotype 1a	‐	14 (87.5)	14 (77.7)	0.60
Genotype 1b	‐	2 (12.5)	1 (5.55)	
Genotype 3a	‐	‐	2 (11.11)	
Genotype 4	‐	‐	1 (5.55)	
HCV viral load (log_10_ copies)^a^	‐	6.12 (3.14 to 7.54)	6.12 (4.74 to 7.38)	0.97
Liver stiffness (Kpa)^a^	4.50 (4 to 5)	6.02 (4 to 8)^d^	20.5 (12 to 36)^d^	0.008
APRI score	nd	0.57 (0.25 to 1.34)	2.05 (0.42 to 11.6)	0.04
ALT (IU/L)^a^	nd	64.3 (25 to 122)	90.9 (25 to 190)	0.09
AST (IU/L)^a^	nd	50.6 (25 to 95)	94.1 (37 to 209)	0.007
γ‐GT (IU/L)^a^	nd	118.9 (31 to 252)	185.9 (72 to 372)	0.06
Albumin (g/dL)^a^	nd	4.33 (3.9 to 5.1)	4.07 (3.1 to 4.6)	0.09
Platelets (×10^3^/mm^3^)^a^	nd	200.0 (136 to 258)	141.2 (25 to 255)	0.003
Total bilirubin (μg/dL)^a^	nd	0.59 (0.2 to 1.6)	1.30 (0.5 to 2.5)	0.0003
Prothrombin time (%)^a^	nd	96.3 (84.8 to 100)	82.4 (50.9 to 100)	0.007

Time of known HIV/HCV infection is defined as the time from first HIV/HCV diagnosis by serology. ALT, alanine aminotransferase; ART, antiretroviral therapy; AST, aspartate transaminase; d4T, stavudine; ddI, didanosine; IDU, intravenous drug user; IFN/PEGIFN, interferon/pegilated interferon; INSTI, integrase strand transfer inhibitor; nd, not determined; NNRTI, non‐nucleoside reverse transcriptase inhibitors; PI, protease inhibitors; γ‐GT, gamma‐glutamyl transpeptidase.

^a^Mean (range); ^b^number of cases (number/total in %); ^c^ *p*=0.0005 control vs. F4; ^d^ *p*<0.0001 F0/F1 versus F4, ANOVA followed by Tukey′s test.

In this new group of HIV/HCV‐coinfected individuals, we confirmed our previous observations 20 showing that advanced fibrosis subjects displayed the lowest CD4^+^ T‐cell and NK cell frequencies (Table 2). Furthermore, we observed that advanced fibrosis subjects displayed lower frequencies of CD56^dim^ but increased percentage of CD56^bright^ cells than HV, and individuals with minimal fibrosis.

**Table 2:** Frequencies of peripheral blood lymphocyte populations in healthy volunteers and HIV/HCV‐coinfected individuals

Characteristics	Control	F0/F1	F4	*p* value
CD4^+^ T‐cells (%)^a^	43.07 (30.00 to 60.00)	32.80 (18.00 to 46.00)	23.53 (9.00 to 34.00)	0.033^b^, 0.0001^c^, 0.037^d^
NK cells (%)^a^	9.63 (4.03 to 17.35)	8.34 (3.13 to 14.40)	4.16 (1.29 to 10.11)	0.0004^c^, 0.003^d^
CD56 dim	96.56 (92.8 to 99.1)	97.46 (93.5 to 99.2)	87.95 (64.4 to 99.2)	0.01^c^, 0.007^d^
CD56 bright	3.44 (0.87 to 7.18)	2.54 (0.73 to 6.50)	12.05 (1.27 to 35.56)	0.01^c^, 0.007^d^

NK, natural killer.

^a^Mean (range); ^b^control vs. F0/F1, ^c^control vs. F4, ^d^F0/F1 versus F4.

### 3.2 Functionality of NK cells is compromised in individuals with advanced liver fibrosis

**Figure 1:**
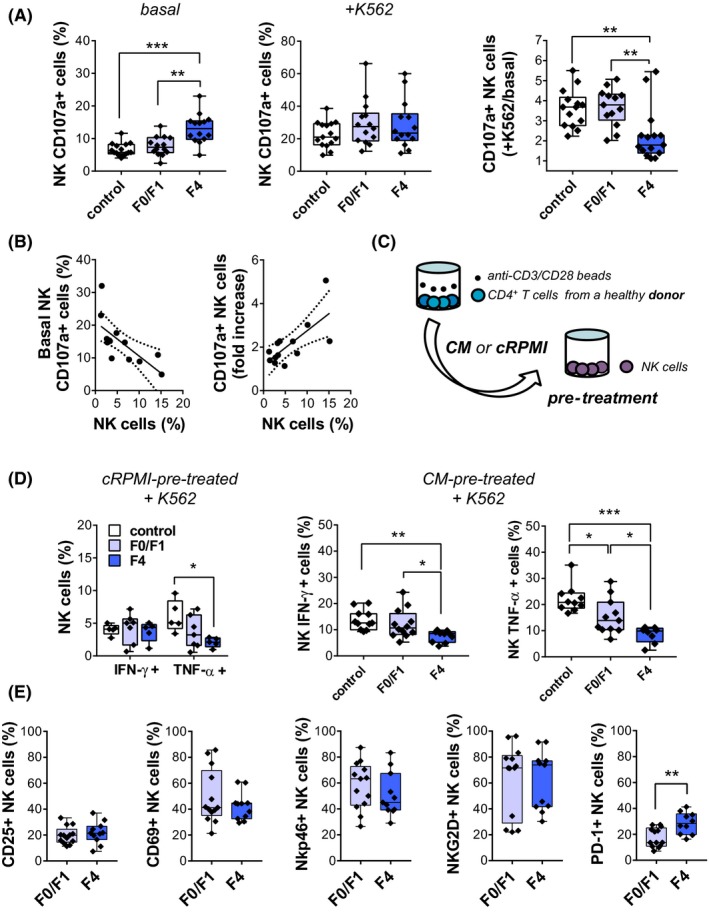
NK cell phenotype and functionality in HIV/HCV‐coinfected individuals with minimal or advanced liver fibrosis (**A**) CD107a externalization in PBMC from control and HIV/HCV‐coinfected individuals with METAVIR F0/F1 or F4 scores incubated with cRPMI (basal) or K562 cells (+K562). (**B**) Correlation between frequency of NK cells and degranulation capacity in F4 individuals. (**C** and **D**) For IFN‐γ and TNF‐α expression, NK cells were pretreated with conditioned medium (CM) from CD4+ T lymphocytes or cRPMI, and subsequently exposed to K562 cells. (**E**) PBMCs were stained for CD25, CD69, NKp46, NKG2D and PD‐1 markers. Individual NK cell frequencies, median and 25th and 75th percentiles are indicated. Statistical comparisons were performed using Kruskal‐Wallis followed by Dunn′s multiple‐comparison tests (**A** and **D**) and Mann‐Whitney test (**E**). NK, natural killer.

First, functionality of NK cells from HV, as well as HIV/HCV‐coinfected individuals was analysed. To evaluate NK cell degranulation, we analysed the expression of cell surface CD107a, as a measure of NK cell degranulation 26. Interestingly, basal (unstimulated) expression of CD107a differed between groups, with the highest percentage of CD107a^+^ NK cells corresponding to F4 individuals (Figure 1A, left panel). However, after K562 coincubation, CD107a expression on NK cells reached similar levels in HIV/HCV‐coinfected individuals and HV (Figure 1A, middle panel), suggesting an impaired ability of NK cells from F4 individuals to be activated *ex vivo*. In fact, the degranulation response (fold change) induced upon incubation of NK cells with K562 cells was significantly lower in F4 individuals compared with the other two groups (Figure 1A, right panel). Interestingly, when F4 individuals were analysed, the frequency of NK cells negatively correlated with basal percentages of CD107a^+^ NK cells (Spearman rho = −0.70, *p* = 0.0007), and positively, with the increased degranulation response induced by K562 cells (Spearman rho = 0.71, *p* = 0.0006) (Figure 1B).

Then, we analysed the pattern of cytokine secretion by NK cells. In order to resemble physiologic activation of NK cells by CD4^+^ T lymphocytes, cytokine secretion by NK cells from both healthy and coinfected individuals was stimulated upon pre‐incubation with CM obtained from CD4^+^ T‐cells activated by anti‐CD3/CD28 coated beads, as described in Figure 1C. Control experiments performed with a HV showed that this treatment effectively activated CD4^+^ T‐cells, increasing the expression of CD69 and the production of a number of cytokines, and also that incubation of NK cells with the CM obtained from activated CD4^+^ T‐cells efficiently activated NK cells, as indicated by the increased expression of CD107a (Figure S1). We next analysed the production of IFN‐γ and TNF‐α by NK cells from all the enrolled groups after incubation with CMs obtained from activated CD4^+^ T‐cells isolated from a HV. In the absence of CM pretreatment, exposure to K562 cells induced low levels of cytokine secretion. While production of IFN‐γ did not differ among groups, TNF‐α secretion was greatly impaired in subjects with advanced fibrosis (Figure 1D). In CM pretreated NK cells, the frequency of IFN‐γ^+^ or TNF‐α^+^ cells was significantly lower in F4 individuals compared with healthy and F0/F1 individuals.

Due to small NK cell frequencies displayed by individuals with advanced fibrosis, effector functions of CD56^dim^ and CD56^bright^ populations could not be studied in all recruited subjects. When analysing degranulation and both IFN‐γ and TNF‐α secretion on CD56^dim^ population; we found similar results as the obtained with total NK cells. Regarding CD56^bright^ cells, while CD107a expression was not significantly affected (Figure S3A), there is a tendency towards a reduction in cytokine production in METAVIR F4 subjects (Figure S3B,C).

To further characterize NK cell populations in HIV/HCV‐coinfected subjects, the expression of the activation markers CD25 and CD69, the activating receptors NKp46 and NKG2D, and PD‐1, a marker of cellular exhaustion, were assessed on recently thawed PBMC (Figure 1E). While the expression of PD‐1 on NK cells was significantly higher in individuals with F4 scores, the other surface markers were not differentially expressed in HIV/HCV‐coinfected individuals. Similar results were obtained analysing both the percentages of NK cells expressing each marker (Figure 1E) and the median fluorescent intensity (MFI) for each of the markers analysed (not shown). Neither functional nor phenotypical differences were found in NK cells according gender (Table S2).

### 3.3 Activation of CD4^+^ T‐cells is similarly induced in HIV/HCV‐coinfected individuals with different stages of hepatic fibrosis

**Figure 2:**
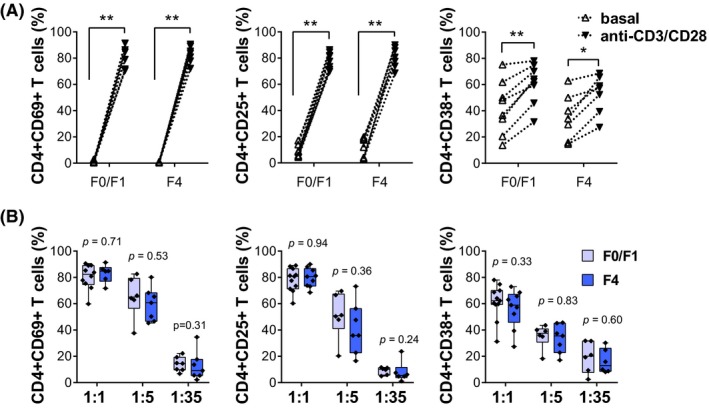
Activation of CD4+ T‐cells from HIV/HCV‐coinfected individuals with minimal or advanced liver fibrosis (**A**) CD69, CD25 and CD38 expression were measured on CD4+ T‐cells from HIV/HCV‐coinfected subjects with METAVIR F0/F1 or F4 scores after 48 hours of vehicle (basal) or anti‐CD3/CD28 stimulation (bead‐to‐cell ratio of 1:1). (**B**) The same markers were determined after anti‐CD3/CD28 stimulation (1:5 or 1:35 ratios; 24 hours). Individual CD4+ T‐cell frequencies, median and 25th and 75th percentiles are indicated. Statistical comparisons were performed using Wilcoxon matched paired test (**A**) and Mann‐Whitney test (**B**).

**Figure 3:**
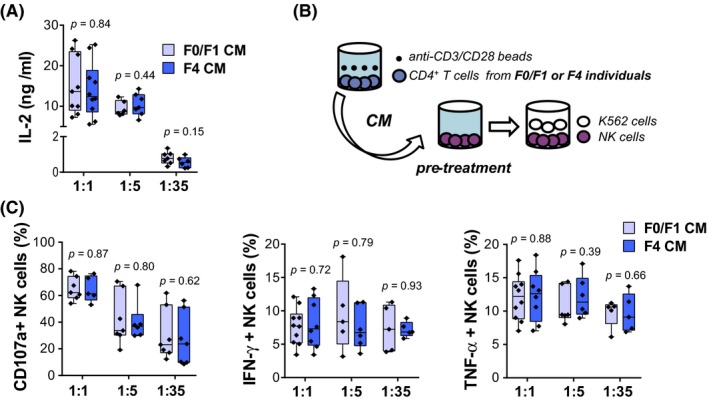
*Ex vivo* effect of CD4+ T‐cells from HIV/HCV‐coinfected individuals with minimal or advanced liver fibrosis on NK cells (**A**) IL‐2 levels in CM. (**B**) Conditioned medium (CM) from activated CD4+ T‐cells from HIV/HCV‐coinfected subjects with METAVIR F0/F1 or F4 scores were collected, and subsequently used for pretreatment of NK cells from one healthy individual. Finally, NK cells were coincubated with K562 cells. **(C)** CD107a, IFN‐γ and TNF‐α expression on NK cells. Individual CD4+ T‐cell frequencies, median and 25th and 75th percentiles are indicated. Statistical comparisons were performed using Mann‐Whitney test. NK, natural killer.

Next, activation of CD4^+^ T‐cells from HIV/HCV‐coinfected individuals was studied. Isolated CD4^+^ T‐cells were stimulated with anti‐CD3/CD28 beads and frequencies of CD4^+^/CD69^+^, CD4^+^/CD25^+^ and CD4^+^/CD38^+^ cells were measured. After 48 hours of stimulation at a bead‐to‐cell ratio of 1:1, frequency of CD4^+^/CD25^+^, CD4^+^/CD69^+^ and CD4^+^/CD38^+^ T‐cells were increased in a similar way in both METAVIR F0/F1 and F4 individuals (Figure 2A). Further experiments were performed using a shorter incubation period and different bead‐to‐cell ratios in order to reveal potential differences between the two groups. No differences were observed between HIV and HCV‐coinfected individuals with minimal or advanced liver fibrosis in terms of frequency of activated CD4^+^ T‐cells (Figure 2B). Similar results were obtained analysing MFI (not shown). Additionally, IL‐2 levels in CD4^+^ T‐cell CM were evaluated by ELISA. In line with the previous results, there were no significant differences between the groups regarding IL‐2 secretion at any tested condition (Figure 3A). Also, CD4^+^ T‐cell activation did not vary between male and female subjects (Table S2). We conclude that there are no differences in the activation pattern of CD4^+^ T‐cells obtained from individuals with minimal or advanced fibrosis.

### 3.4 NK cell functionality is similarly modulated by CD4^+^ T‐cells purified from individuals with minimal or advanced fibrosis

Finally, *ex vivo* stimulation of NK cells by cytokines from activated CD4^+^ T‐cells of HIV/HCV‐coinfected individuals was evaluated. NK cells from a selected HV were pretreated with CM from activated CD4^+^ T‐cells (Figure 3B). Subsequently, NK cells were coincubated with K562 cells, and CD107a externalization, as well as cytokine production was measured. As shown in Figure 3C, induction of NK cell degranulation progressively decreased, as cells were pre‐incubated with CM from CD4^+^ T‐cells stimulated with increasing cell‐to‐anti‐CD3/CD28 bead ratios, probably reflecting a decline in cytokine concentration, for example, IL‐2 (Figure 3A), in CD4^+^ T‐cell CM. However, no significant differences regarding CD107a expression on healthy NK cells were registered after treatment with CM from CD4^+^ T‐cells of F0/F1 or F4 individuals, at any tested condition. Cytokine secretion was similarly upregulated regardless of the strength of CD4^+^ T‐cell activation. In line with the previous degranulation results, fibrosis status of individuals was not differentially associated to either IFN‐γ or TNF‐α production by NK cells. MFI evaluation of degranulation and cytokine secretion yielded similar results (not shown).

In summary, we did not observe differences in the capacity of CD4^+^ T‐cells from individuals with minimal or advanced fibrosis, to modulate neither NK cell degranulation or IFN‐γ and TNF‐α secretion.

## 4 Discussion

Progression of liver disease in HCV‐monoinfected and HIV/HCV‐coinfected individuals is extremely variable. In our patient cohort, HIV/HCV‐coinfected individuals had similar time of known HIV and HCV infection and ART, however, some of them progressed to cirrhosis whereas others had minimal liver fibrosis. NK cells have shown to play an important role in the immune response against HCV infection and in the modulation of liver fibrosis development. It was previously reported in HCV monoinfection and HIV/HCV coinfection that peripheral NK cell frequency is significantly decreased 27, 28, 29. Recently, we showed that among HIV/HCV‐coinfected subjects, advanced fibrosis was associated to the lowest frequencies of circulating NK cells 20. Although it has been suggested that NK cells were preferentially localized in liver tissue during viral chronic infections 30, mechanisms for decreased peripheral NK cell frequencies in individuals with advanced fibrosis are not fully understood. In the present study, we report that the compartment of NK cells in patients with advanced fibrosis shows not only a quantitative defect but also a compromise in cell functionality.

In this study, we show that *ex vivo* degranulation of NK cells from cirrhotic individuals coinfected with HIV and HCV is poorly induced by K562 cells, perhaps reflecting an exhausted phenotype. Augmented levels of basal degranulation were described in antiretroviral‐treated HIV‐positive individuals 31, possibly indicating the presence of targets for NK cell‐mediated natural cytotoxicity in the course of infection. HCV‐associated immune response, and also both hepatic and systemic proinflammatory milieu may contribute to this phenomenon. Interestingly, here we show that cirrhotic patients with the lowest NK cell frequencies displayed the greatest percentages of basal CD107a+ NK cells, and the weakest induction of NK cell degranulation by K562 cells. This observation indicates that the frequency and function of NK cells could be related, although the mechanisms underlying this association remain to be defined. Our results suggest that in HIV/HCV‐coinfected individuals, liver damage may lead to *in vivo* over‐activation and functional exhaustion (i.e. the impossibility to exhibit a significant degranulation upon exposure to susceptible targets) of peripheral NK cell subset.

Regarding cytokine secretion, we show that IFN‐γ and TNF‐α production by NK cells is significantly impaired in individuals with advanced fibrosis. In particular, our results may indicate that NK cells from F4 individuals failed at responding to CD4^+^ T‐cell derived stimuli. Nevertheless, since NK cells were studied in PBMC fractions, interaction with other immune cells and their products may have influenced the observed results. It is evident that experimental settings, for example, cytokine prestimulation of NK cells, evaluation of spontaneous degranulation, and interaction with other immune cells are critical when analysing *ex vivo* NK cell cytotoxicity. Together, our results are consistent with those reporting impaired functionality of NK cells during HCV monoinfection 32, 33, 34, 35, 36, and also HIV/HCV coinfection 29. However, they could be also interpreted in the context of a functional NK cell dichotomy, where NK cells display a decreased production of antiviral cytokines but an enhanced basal cytotoxicity, which is impaired when these cells are exposed to their targets 18, 37. Cytokine secretion and cytotoxicity can be uncoupled when NK cells contact their target cells 38.

Possible mechanisms underlying a compromised NK cell function in the course of HCV infection have been related to changes in the activation state and/or an imbalanced NK cell receptor expression 39. Here, we evaluated CD69, CD25, NKp46 and NKp30 cell markers but only PD‐1 expression was associated to liver fibrosis progression. In line with the results from functional assays, PD‐1 expression may be reflecting an exhausted NK pool in individuals with advanced liver fibrosis. Although PD‐1 expression in NK cells was already linked to chronic HCV infection 40, to the best of our knowledge, no relationship between advanced liver fibrosis and the PD‐1/PDL‐1 axis in NK cells has been previously addressed. Further studies are required to analyse the utility of PD‐1 as a marker of fibrosis progression as well as to explore whether PD‐1 could represent a potential immune check point for targeted therapies aimed to modulate liver fibrosis.

Despite all individuals were on successful ART therapy, F4 individuals have lower CD4^+^ T‐cell counts and percentages than subjects with minimal fibrosis. Studies of liver fibrosis progression in HIV seronegative individuals have proposed that low absolute CD4^+^ T‐cell counts may be attributable to advanced liver disease, due to hypersplenism secondary to portal hypertension, and consequently, leucopenia 41, 42, 43. Nevertheless, hypersplenism could not be associated with the decrease in the percentage CD4^+^ T‐cell observed in our group of cirrhotic individuals (since leucopenia affects the absolute number of the different leucocytes but not the proportion of them). Therefore, our results suggest that the lower frequency of these cells could be associated with the progression to liver fibrosis rather than being a consequence of end stage liver disease. It is well known that CD4^+^ T‐cells boost NK cell activation via IL‐2 secretion 44 and recently, impaired CD4^+^ T‐cell stimulation of NK cells has been linked to liver fibrosis by comparing HV and HIV/HCV‐coinfected individuals, 45. Our present observations differ from this study in two ways. First, we did not analyse coinfected subjects as a unique population, but rather we stratified coinfected individuals according to their degree of fibrosis, enabling us to define associations between the degree of fibrosis and the phenotype and function of NK cells. Second, we observed that the response to activation stimuli of CD4^+^ T‐cells in coinfected subjects as well as their ability to modulate the function of NK cells did not differ between individuals with minimal and maximal degree of fibrosis. It has been reported that HIV/HCV‐coinfected subjects with higher chances to develop liver fibrosis are those with lower CD4^+^ T‐cell recovery after HIV treatment 46. In view of our results, CD4^+^ T‐cell count might contribute more significantly to fibrosis progression than a deficient CD4^+^ T‐cell modulation capacity.

Our study has some limitations. First, a larger sample size would permit stronger associations between variables, and especially to robustly address NK CD56^bright^ population. Also, it would be very interesting to study CD56^neg^ NK cells, since they are described as an aberrant and hypo functional cell subset found at elevated levels in individuals chronically infected with HIV‐1 and HCV 47. Finally, although the main focus of our study was to determine whether CD4^+^ T‐cells from coinfected individuals with minimal or advanced fibrosis had different capacity to stimulate NK cells, we cannot rule out that a defect in the function of HIV‐ or HCV‐specific CD4^+^ T‐cells might contribute to NK cell dysfunction in coinfected subjects. It would be very interesting to investigate the potential role of HIV‐ or HCV‐specific CD4^+^ T‐cells to modulate liver fibrosis.

## 5 Conclusions

Association between NK cell phenotype and function and the outcome of acute HCV infection was extensively reported 48, 49, 50, nevertheless, it remains unclear whether phenotypical and/or functional characteristics of NK cells are actually determinants for the rate of liver fibrosis progression. We and others have previously shown an association between low NK and CD4^+^ T‐cell counts with advanced liver fibrosis in HIV/HCV‐coinfected individuals 20, 29. Here, we provide further support for this phenomenon, and also demonstrate that NK functionality is significantly impaired in coinfected individuals with advanced fibrosis. Although the underlying mechanisms remain to be defined, our results suggest they could not be attributed to an inefficient modulation of NK cell function by CD4^+^ T‐cells. In sum, lower CD4^+^ T and NK cells, as well as a decreased NK functionality may represent *ex vivo* biological markers of advanced liver disease.

## Competing interests

No conflicts of interest including financial and other relationships are declared.

## Authors’ contributions

MLP, NLL, PEC and GJT contributed to study conception and design of experiments. MLP, NLL, GP, AES, MJR and AM contributed to data acquisition. MLP, NLL, JPS, YAG, AU, DSO and GJT contributed to data analysis. MLP, NLL, YAG, PEC and GJT contributed to writing, reviewing and final approval of article.

## Supporting information


**Figure S1.** Lymphocytes isolated from a selected healthy volunteer. NK and CD4^+^ T‐cells were purified from PBMC of a 42‐year‐old woman, HIV/HCV/HBV negative, with no history of alcohol consumption, tabaquism or ilicit drug use, thyroid or celiac disease, and no other clinically relevant conditions. % NK cells: 8.62, CD4^+^ T‐cell count: 754, % CD4^+^ T‐cells: 38. (**A**) CD4^+^ T‐cells were stimulated with anti‐CD3/CD28 beads in different bead‐to‐cell ratios (1:1, 48 hours; others 24 hours), and percentages of CD69+/CD4^+^ T cells were monitored. IL‐2 levels were also measured in corresponding culture supernatants. Right: Additional cytokines were quantified in CD4^+^ T‐cell CM (1:1, 48 hours). (**B**) CD107a externalization in CM‐prestimulated PBMC, co‐cultured with K562 cells (CM+K562). As control, cRPMI‐prestimulated PBMC were either exposed to K562 cells (+K562) or cRPMI (basal). Determinations were performed in duplicate. Data are presented as mean ± SD. Statistical comparisons were performed using Wilcoxon matched paired test. nd, not detected.

**Figure S2.** Gating strategy, and representative dot plots for flow cytometry analysis. (**A**) NK cell subset was defined as CD3‐/CD56+ viable lymphocytes. (**B**) Upon incubation with cRPMI (basal) or K562 cells (+K562), CD107a+ NK cells were determined (Top). Intracellular IFN‐γ and TNF‐α expression was measured in cRPMI or CM‐prestimulated NK cells, co‐cultured with K562 cells (Bottom). (**C**) CD69+, CD25+ or CD38+ cells were determined in vehicle (control) or anti‐CD3/CD28‐stimulated CD4^+^ T‐cells. At least one thousand events were acquired for both NK and CD4^+^ T‐cell gates.

**Figure S3.** Evaluation of NK cell effector functions in CD56 dim and bright populations. (**A**) PBMCs from healthy and HIV/HCV‐coinfected individuals with METAVIR F0/F1 or F4 scores were incubated with cRPMI (basal) or K562 cells (+K562). Fold change induction in CD107a expression (+K562/basal) was evaluated in CD56^dim^ and CD56^bright^ cell subsets. (**B** and **C**) For cytokine expression, PBMCs cells were pretreated with conditioned medium from CD4^+^ T‐lymphocytes, and subsequently exposed to K562 cells. Frequencies of IFN‐γ **(B)** and TNF‐α‐positive cells (**C**) were determined in CD56^dim^ and CD56^bright^ cell subsets. Statistical analysis was performed using Kruskal‐Wallis followed by Dunn′s multiple‐comparison.

**Table S1.** Fluorochrome‐conjugated antibody panels.
**Table S2.** Differences in NK and CD4^+^ T‐cell phenotypic and functional markers according gender.

## References

[1] World Health Organization . Global hepatitis report. 2017.

[2] Platt L , Easterbrook P , Gower E , McDonald B , Sabin K , McGowan C , et al. Prevalence and burden of HCV co‐infection in people living with HIV: a global systematic review and meta‐analysis. Lancet Infect Dis. 2016;16(7):797–808. 26922272 10.1016/S1473-3099(15)00485-5

[3] Operskalski EA , Kovacs A . HIV/HCV co‐infection: pathogenesis, clinical complications, treatment, and new therapeutic technologies. Curr HIV/AIDS Rep. 2011;8(1):12–22. 21221855 10.1007/s11904-010-0071-3 PMC3035774

[4] Ioannou GN , Bryson CL , Weiss NS , Miller R , Scott JD , Boyko EJ . The prevalence of cirrhosis and hepatocellular carcinoma in patients with human immunodeficiency virus infection. Hepatology. 2013;57(1):249–57. 22532055 10.1002/hep.25800

[5] Lin W , Weinberg EM , Chung RT . Pathogenesis of accelerated fibrosis in HIV/HCV co‐infection. J Infect Dis. 2013;207 Suppl 1:S13–8. 23390300 10.1093/infdis/jis926 PMC3611768

[6] Tosello‐Trampont A , Surette FA , Ewald SE , Hahn YS . Immunoregulatory Role of NK Cells in Tissue Inflammation and Regeneration. Front Immunol. 2017;8:301. 28373874 10.3389/fimmu.2017.00301 PMC5357635

[7] Fasbender F , Widera A , Hengstler JG , Watzl C . Natural Killer Cells and Liver Fibrosis. Front Immunol. 2016;7:19. 26858722 10.3389/fimmu.2016.00019 PMC4731511

[8] Radaeva S , Sun R , Jaruga B , Nguyen VT , Tian Z , Gao B . Natural killer cells ameliorate liver fibrosis by killing activated stellate cells in NKG2D‐dependent and tumor necrosis factor‐related apoptosis‐inducing ligand‐dependent manners. Gastroenterology. 2006;130(2):435–52. 16472598 10.1053/j.gastro.2005.10.055

[9] Melhem A , Muhanna N , Bishara A , Alvarez CE , Ilan Y , Bishara T , et al. Anti‐fibrotic activity of NK cells in experimental liver injury through killing of activated HSC. J Hepatol. 2006;45(1):60–71. 16515819 10.1016/j.jhep.2005.12.025

[10] Muhanna N , Abu Tair L , Doron S , Amer J , Azzeh M , Mahamid M , et al. Amelioration of hepatic fibrosis by NK cell activation. Gut. 2011;60(1):90–8. 20660699 10.1136/gut.2010.211136

[11] Gur C , Doron S , Kfir‐Erenfeld S , Horwitz E , Abu‐Tair L , Safadi R , et al. NKp46‐mediated killing of human and mouse hepatic stellate cells attenuates liver fibrosis. Gut. 2012;61(6):885–93. 22198715 10.1136/gutjnl-2011-301400

[12] Kramer B , Korner C , Kebschull M , Glassner A , Eisenhardt M , Nischalke HD , et al. Natural killer p46High expression defines a natural killer cell subset that is potentially involved in control of hepatitis C virus replication and modulation of liver fibrosis. Hepatology. 2012;56(4):1201–13. 22532190 10.1002/hep.25804

[13] Glassner A , Eisenhardt M , Kramer B , Korner C , Coenen M , Sauerbruch T , et al. NK cells from HCV‐infected patients effectively induce apoptosis of activated primary human hepatic stellate cells in a TRAIL‐, FasL‐ and NKG2D‐dependent manner. Lab Invest. 2012;92(7):967–77. 22449797 10.1038/labinvest.2012.54

[14] Eisenhardt M , Glassner A , Kramer B , Korner C , Sibbing B , Kokordelis P , et al. The CXCR3(+)CD56Bright phenotype characterizes a distinct NK cell subset with anti‐fibrotic potential that shows dys‐regulated activity in hepatitis C. PLoS ONE. 2012;7(7):e38846. 22792160 10.1371/journal.pone.0038846 PMC3390318

[15] Gao B , Radaeva S . Natural killer and natural killer T cells in liver fibrosis. Biochem Biophys Acta. 2013;1832(7):1061–9. 23022478 10.1016/j.bbadis.2012.09.008 PMC3552008

[16] Jeong WI , Park O , Suh YG , Byun JS , Park SY , Choi E , et al. Suppression of innate immunity (natural killer cell/interferon‐gamma) in the advanced stages of liver fibrosis in mice. Hepatology. 2011;53(4):1342–51. 21480338 10.1002/hep.24190 PMC3079530

[17] Shi J , Zhao J , Zhang X , Cheng Y , Hu J , Li Y , et al. Activated hepatic stellate cells impair NK cell anti‐fibrosis capacity through a TGF‐beta‐dependent emperipolesis in HBV cirrhotic patients. Sci Rep. 2017;7:44544. 28291251 10.1038/srep44544 PMC5349579

[18] Rehermann B . Natural killer cells in viral hepatitis. Cell Mol Gastroenterol Hepatol. 2015;1(6):578–88. 26682281 10.1016/j.jcmgh.2015.09.004 PMC4678927

[19] Mikulak J , Oriolo F , Zaghi E , Di Vito C , Mavilio D . Natural killer cells in HIV‐1 infection and therapy. AIDS. 2017;31(17):2317–30. 28926399 10.1097/QAD.0000000000001645 PMC5892189

[20] Laufer N , Ojeda D , Polo ML , Martinez A , Perez H , Turk G , et al. CD4(+) T cells and natural killer cells: biomarkers for hepatic fibrosis in human immunodeficiency virus/hepatitis C virus‐coinfected patients. World J Hepatol. 2017;9(25):1073–80. 28951779 10.4254/wjh.v9.i25.1073 PMC5596314

[21] Castera L , Vergniol J , Foucher J , Le Bail B , Chanteloup E , Haaser M , et al. Prospective comparison of transient elastography, Fibrotest, APRI, and liver biopsy for the assessment of fibrosis in chronic hepatitis C. Gastroenterology. 2005;128(2):343–50. 15685546 10.1053/j.gastro.2004.11.018

[22] Bedossa P , Poynard T . An algorithm for the grading of activity in chronic hepatitis C. The METAVIR Cooperative Study Group. Hepatology. 1996;24(2):289–93. 8690394 10.1002/hep.510240201

[23] Sanchez‐Conde M , Montes‐Ramirez ML , Miralles P , Alvarez JM , Bellon JM , Ramirez M , et al. Comparison of transient elastography and liver biopsy for the assessment of liver fibrosis in HIV/hepatitis C virus‐coinfected patients and correlation with noninvasive serum markers. J Viral Hepatitis. 2010;17(4):280–6. 10.1111/j.1365-2893.2009.01180.x 19732322

[24] Baden R , Rockstroh JK , Buti M . Natural history and management of hepatitis C: does sex play a role? J Infect Dis. 2014;209 Suppl 3:S81–5. 24966194 10.1093/infdis/jiu057

[25] Collazos J , Carton JA , Asensi V . Gender differences in liver fibrosis and hepatitis C virus‐related parameters in patients coinfected with human immunodeficiency virus. Curr HIV Res. 2011;9(5):339–45. 21827383 10.2174/157016211797635982

[26] Alter G , Malenfant JM , Altfeld M . CD107a as a functional marker for the identification of natural killer cell activity. J Immunol Methods. 2004;294(1–2):15–22. 15604012 10.1016/j.jim.2004.08.008

[27] Meier UC , Owen RE , Taylor E , Worth A , Naoumov N , Willberg C , et al. Shared alterations in NK cell frequency, phenotype, and function in chronic human immunodeficiency virus and hepatitis C virus infections. J Virol. 2005;79(19):12365–74. 16160163 10.1128/JVI.79.19.12365-12374.2005 PMC1211534

[28] Morishima C , Paschal DM , Wang CC , Yoshihara CS , Wood BL , Yeo AE , et al. Decreased NK cell frequency in chronic hepatitis C does not affect *ex vivo* cytolytic killing. Hepatology. 2006;43(3):573–80. 16496327 10.1002/hep.21073

[29] Kaczmarek DJ , Kokordelis P , Kramer B , Glassner A , Wolter F , Goeser F , et al. Alterations of the NK cell pool in HIV/HCV co‐infection. PLoS ONE. 2017;12(4):e0174465. 28380039 10.1371/journal.pone.0174465 PMC5381812

[30] Zheng Q , Zhu YY , Chen J , Ye YB , Li JY , Liu YR , et al. Activated natural killer cells accelerate liver damage in patients with chronic hepatitis B virus infection. Clin Exp Immunol. 2015;180(3):499–508. 25639451 10.1111/cei.12597 PMC4449778

[31] Lichtfuss GF , Cheng WJ , Farsakoglu Y , Paukovics G , Rajasuriar R , Velayudham P , et al. Virologically suppressed HIV patients show activation of NK cells and persistent innate immune activation. J Immunol. 2012;189(3):1491–9. 22745371 10.4049/jimmunol.1200458

[32] Sene D , Levasseur F , Abel M , Lambert M , Camous X , Hernandez C , et al. Hepatitis C virus (HCV) evades NKG2D‐dependent NK cell responses through NS5A‐mediated imbalance of inflammatory cytokines. PLoS Pathog. 2010;6(11):e1001184. 21085608 10.1371/journal.ppat.1001184 PMC2978723

[33] Varchetta S , Mele D , Mantovani S , Oliviero B , Cremonesi E , Ludovisi S , et al. Impaired intrahepatic natural killer cell cytotoxic function in chronic hepatitis C virus infection. Hepatology. 2012;56(3):841–9. 22431186 10.1002/hep.25723

[34] Pembroke T , Christian A , Jones E , Hills RK , Wang EC , Gallimore AM , et al. The paradox of NKp46 + natural killer cells: drivers of severe hepatitis C virus‐induced pathology but in‐vivo resistance to interferon alpha treatment. Gut. 2014;63(3):515–24. 23665989 10.1136/gutjnl-2013-304472 PMC3932740

[35] Lunemann S , Malone DF , Hengst J , Port K , Grabowski J , Deterding K , et al. Compromised function of natural killer cells in acute and chronic viral hepatitis. J Infect Dis. 2014;209(9):1362–73. 24154737 10.1093/infdis/jit561

[36] Podhorzer A , Dirchwolf M , Machicote A , Belen S , Montal S , Paz S , et al. The clinical features of patients with chronic hepatitis C virus infections are associated with killer cell immunoglobulin‐like receptor genes and their expression on the surface of natural killer cells. Front Immunol. 2017;8:1912. 29354127 10.3389/fimmu.2017.01912 PMC5760500

[37] Mondelli MU , Oliviero B , Mele D , Mantovani S , Gazzabin C , Varchetta S . Natural killer cell functional dichotomy: a feature of chronic viral hepatitis? Front Immunol. 2012;3:351. 23420385 10.3389/fimmu.2012.00351 PMC3572686

[38] Rajasekaran K , Kumar P , Schuldt KM , Peterson EJ , Vanhaesebroeck B , Dixit V , et al. Signaling by Fyn‐ADAP via the Carma1‐Bcl‐10‐MAP3K7 signalosome exclusively regulates inflammatory cytokine production in NK cells. Nat Immunol. 2013;14(11):1127–36. 24036998 10.1038/ni.2708 PMC3855032

[39] Gardiner CM . NK cell function and receptor diversity in the context of HCV infection. Front Microbiol. 2015;6:1061. 26483779 10.3389/fmicb.2015.01061 PMC4588102

[40] Collister M , Ellison C , Li Q , Minuk GY , Rempel JD , Kung SK . The influence of hepatitis C viral loads on natural killer cell function. Gastroenterol Res. 2019;12(1):8–15. 10.14740/gr1081w PMC6396790 30834029

[41] McGovern BH , Golan Y , Lopez M , Pratt D , Lawton A , Moore G , et al. The impact of cirrhosis on CD4+ T cell counts in HIV‐seronegative patients. Clin Infect. 2007;44(3):431–7. 10.1086/509580 17205454

[42] Sullivan T . The implications of low absolute CD4 counts in patients with cirrhosis and human immunodeficiency virus. Open Forum Infect Dis. 2016;3(2):ofw060. 27419148 10.1093/ofid/ofw060 PMC4943555

[43] Albillos A , Lario M , Alvarez‐Mon M . Cirrhosis‐associated immune Dysfunction: distinctive features and clinical relevance. J Hepatol. 2014;61(6):1385–96. 25135860 10.1016/j.jhep.2014.08.010

[44] Kerdiles Y , Ugolini S , Vivier E . T cell regulation of natural killer cells. J Exp Med. 2013;210(6):1065–8. 23733834 10.1084/jem.20130960 PMC3674696

[45] Glassner A , Eisenhardt M , Kokordelis P , Kramer B , Wolter F , Nischalke HD , et al. Impaired CD4(+) T cell stimulation of NK cell anti‐fibrotic activity may contribute to accelerated liver fibrosis progression in HIV/HCV patients. J Hepatol. 2013;59(3):427–33. 23665286 10.1016/j.jhep.2013.04.029

[46] Dharan NJ , Neuhaus J , Rockstroh JK , Peters L , Gordin F , Arenas‐Pinto A , et al. Benefit of early versus deferred antiretroviral therapy on progression of liver fibrosis among people with HIV in the START randomized trial. Hepatology. 2019;69(3):1135–50. 30298608 10.1002/hep.30296 PMC6393919

[47] Bjorkstrom NK , Ljunggren HG , Sandberg JK . CD56 negative NK cells: origin, function, and role in chronic viral disease. Trends Immunol. 2010;31(11):401–6. 20829113 10.1016/j.it.2010.08.003

[48] Khakoo SI , Thio CL , Martin MP , Brooks CR , Gao X , Astemborski J , et al. HLA and NK cell inhibitory receptor genes in resolving hepatitis C virus infection. Science. 2004;305(5685):872–4. 15297676 10.1126/science.1097670

[49] Alter G , Jost S , Rihn S , Reyor LL , Nolan BE , Ghebremichael M , et al. Reduced frequencies of NKp30+NKp46+, CD161+, and NKG2D+ NK cells in acute HCV infection may predict viral clearance. J Hepatol. 2011;55(2):278–88. 21168454 10.1016/j.jhep.2010.11.030 PMC3729214

[50] Shoukry NH , Pelletier S , Chang KM . A view to natural killer cells in hepatitis C. Gastroenterology. 2011;141(4):1144–8. 21875586 10.1053/j.gastro.2011.08.025

