# Supplementary figures and images for: Liver cirrhosis in HIV/HCV‐coinfected individuals is related to NK cell dysfunction and exhaustion, but not to an impaired NK cell modulation by CD4+ T‐cells
Source: J Int AIDS Soc. 2019 Sep 19;22(9):e25375. doi: 10.1002/jia2.25375 (PMC6752153; doi:10.1002/jia2.25375)

**A****CD4+ CD69+ T cells (%)**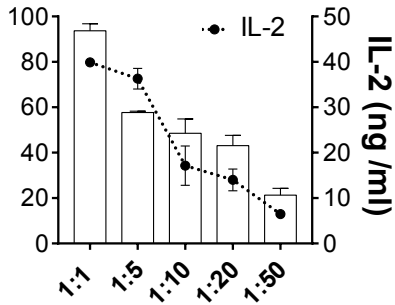**Cytokine (ng/ml)**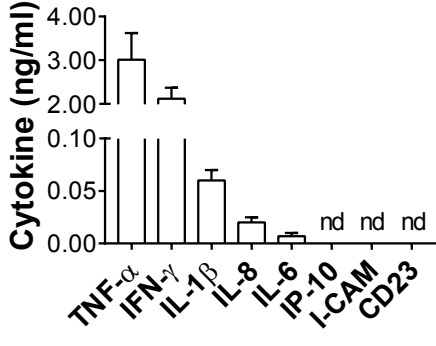**B****CD107a+ NK cells (%)**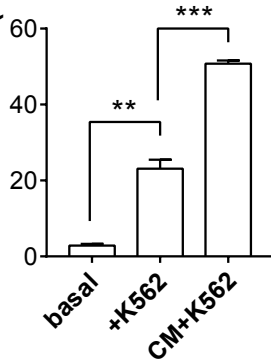

Supplement: Supplementary file 1 — Figure S1. Lymphocytes isolated from a selected healthy volunteer. NK and CD4+ T‐cells were purified from PBMC of a 42‐year‐old woman, HIV/HCV/HBV negative, with no history of alcohol consumption, tabaquism or ilicit drug use, thyroid or celiac disease, and no other clinically relevant conditions. % NK cells: 8.62, CD4+ T‐cell count: 754, % CD4+ T‐cells: 38. (A) CD4+ T‐cells were stimulated with anti‐CD3/CD28 beads in different bead‐to‐cell ratios (1:1, 48 hours; others 24 hours), and percentages of CD69+/CD4+ T cells were monitored. IL‐2 levels were also measured in corresponding culture supernatants. Right: Additional cytokines were quantified in CD4+ T‐cell CM (1:1, 48 hours). (B) CD107a externalization in CM‐prestimulated PBMC, co‐cultured with K562 cells (CM+K562). As control, cRPMI‐prestimulated PBMC were either exposed to K562 cells (+K562) or cRPMI (basal). Determinations were performed in duplicate. Data are presented as mean ± SD. Statistical comparisons were performed using Wilcoxon matched paired test. nd, not detected. [file JIA2-22-e25375-s001.pdf]

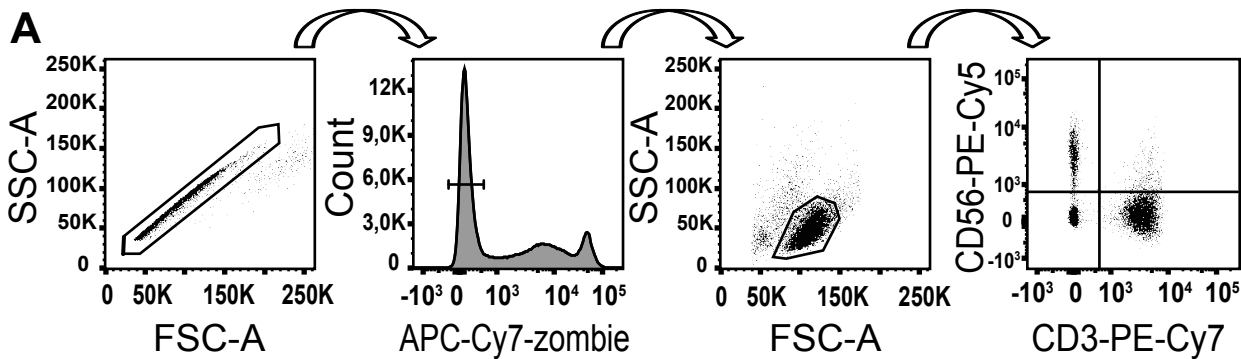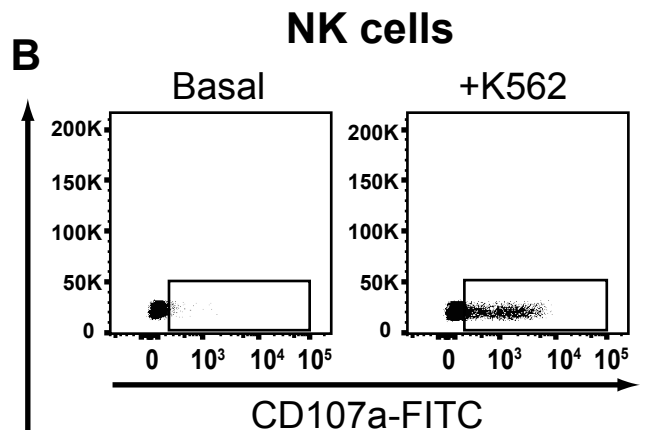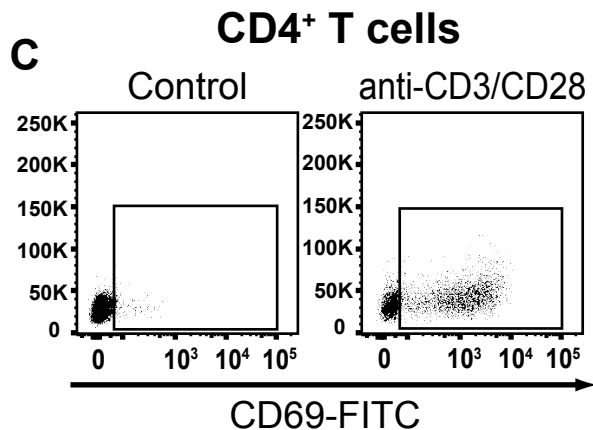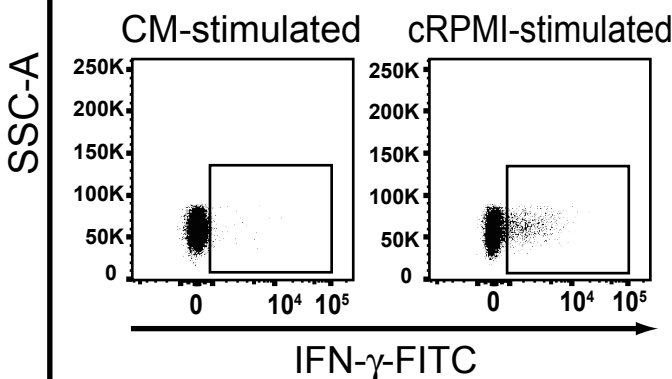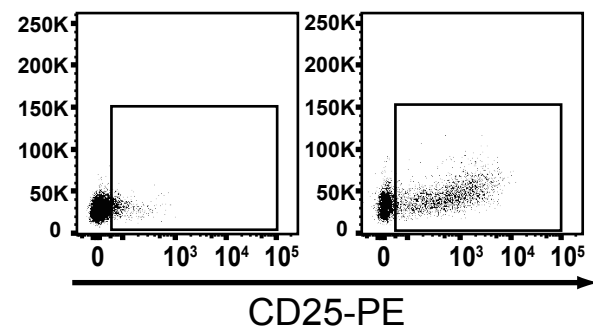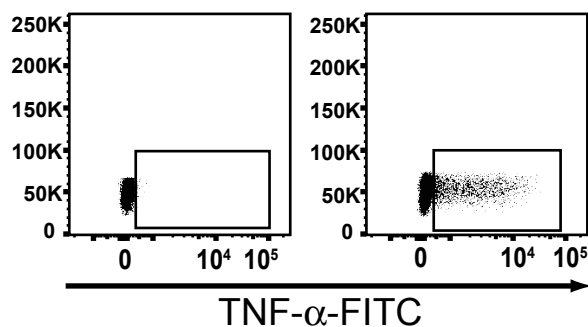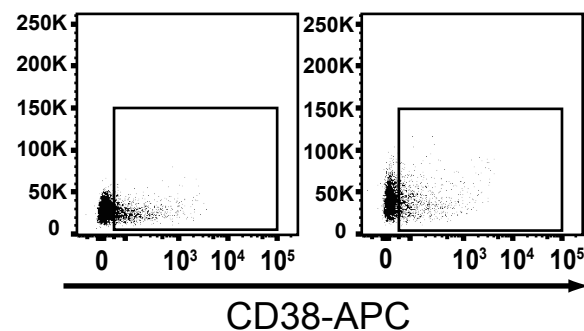

Supplement: Supplementary file 2 — Figure S2. Gating strategy, and representative dot plots for flow cytometry analysis. (A) NK cell subset was defined as CD3‐/CD56+ viable lymphocytes. (B) Upon incubation with cRPMI (basal) or K562 cells (+K562), CD107a+ NK cells were determined (Top). Intracellular IFN‐γ and TNF‐α expression was measured in cRPMI or CM‐prestimulated NK cells, co‐cultured with K562 cells (Bottom). (C) CD69+, CD25+ or CD38+ cells were determined in vehicle (control) or anti‐CD3/CD28‐stimulated CD4+ T‐cells. At least one thousand events were acquired for both NK and CD4+ T‐cell gates. [file JIA2-22-e25375-s002.pdf]

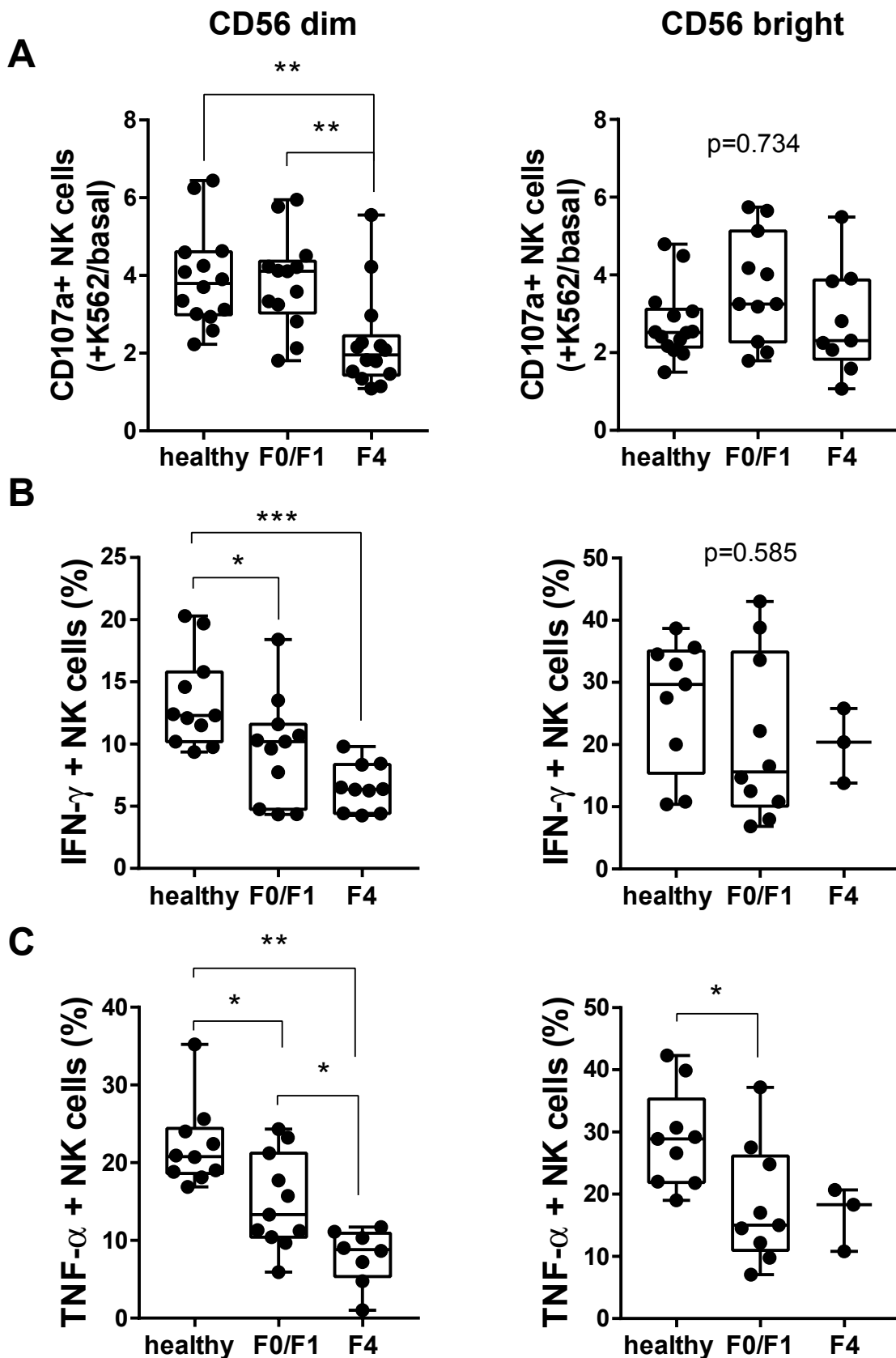

Supplement: Supplementary file 3 — Figure S3. Evaluation of NK cell effector functions in CD56 dim and bright populations. (A) PBMCs from healthy and HIV/HCV‐coinfected individuals with METAVIR F0/F1 or F4 scores were incubated with cRPMI (basal) or K562 cells (+K562). Fold change induction in CD107a expression (+K562/basal) was evaluated in CD56dim and CD56bright cell subsets. (B and C) For cytokine expression, PBMCs cells were pretreated with conditioned medium from CD4+ T‐lymphocytes, and subsequently exposed to K562 cells. Frequencies of IFN‐γ (B) and TNF‐α‐positive cells (C) were determined in CD56dim and CD56bright cell subsets. Statistical analysis was performed using Kruskal‐Wallis followed by Dunn′s multiple‐comparison. [file JIA2-22-e25375-s003.pdf]
