# Supplementary material for: Liver cirrhosis in HIV/HCV‐coinfected individuals is related to NK cell dysfunction and exhaustion, but not to an impaired NK cell modulation by CD4+ T‐cells
Source: J Int AIDS Soc. 2019 Sep 19;22(9):e25375. doi: 10.1002/jia2.25375 (PMC6752153; doi:10.1002/jia2.25375)
Supplement: Supplementary file 4 — Table S1. Fluorochrome‐conjugated antibody panels. Table S2. Differences in NK and CD4+ T‐cell phenotypic and functional markers according gender. [file JIA2-22-e25375-s004.docx]

**Liver cirrhosis in HIV/HCV-coinfected individuals is related to NK cell dysfunction and exhaustion, but not to an impaired NK cell modulation by CD4^+^ T-cells**

María L. Polo, Yanina A. Ghiglione, Jimena P. Salido, Alejandra Urioste, Gabriela Poblete, Alicia E. Sisto, Ana Martinez, María J. Rolón, Diego S. Ojeda, Pedro E. Cahn, Gabriela J. Turk, and Natalia L. Laufer.

**Table of contents:**

| *Isolation of PBMC, NK and CD4+ T-cells……………………………………………………………………………………….* | *1* |
| --- | --- |
| *K562 cell line……………………………………………………………………………………………………………………….* | *1* |
| *Cytokine secretion…………………………………………………………………………………………………………………* | *2* |
| *Statistical analysis…………………………………………………………………………………………………………………* | *2* |
| *Supplemental Tables……………………………………………………………………………………………………………...* | ***2*** |
| *Table S1…………………………………………………………………………………………………………………………….* | *2* |
| *Table S2…………………………………………………………………………………………………………………………….* | *3* |
| *Supplementary Figures…………………………………………………………………………………………………………...* | ***3*** |
| *Fig. S1………………………………………………………………………………………………………………………………* | *3* |
| *Fig. S2………………………………………………………………………………………………………………………………* | *4* |
| *Fig. S3 ……………………………………………………………………………………………………………………………...* | *5* |
| *References………………………………………………………………………………………………………………………….* | *6* |

**Isolation of PBMC, NK and CD4+ T-cells:**

Peripheral blood mononuclear cells (PBMC) were obtained from whole blood by Ficoll-Hypaque centrifugation (GE Healthcare, UK) and cryopreserved at -80°C for up to 4 months. Autologous red blood cells (RBC) were purified and stored in phosphate buffer saline buffer (PBS) at 4°C. NK and CD4^+^ T-cell isolation was achieved by using a modification of the RosetteSep Enrichment Cocktail protocol (Stemcell Technologies, Canada) **(1).** Briefly, 30 million PBMC were co-incubated with RBC (PBMC: RBC ratio of 1:100) in 1 ml of PBS plus 50 μl of RosetteSep Enrichment Cocktail. Cell suspension was then diluted, and NK or CD4+ T-cells were purified on a Ficoll-Hypaque gradient. Purity of isolated cells was >90% as determined by flow cytometry. Cells were cultured in complete RPMI-1640 medium (cRPMI) containing 10% fetal bovine serum, 2 mM l-glutamine, 100 IU/ml penicillin and 100 μg/ml of streptomycin (all reagents, Gibco SRL, USA).

**K562 cell line:**

Three days before the experiments, the chronic myelogenous leukemia K562 cell line was thawed and grown at 37°C and 5% CO2 in cRPMI.

**Cytokine secretion:**

Levels of CD23, I-CAM, IP-10, IL-2, IL-6, IL-8, IL-1β, IFN-γ and TNF-α in CM were determined by ELISA (Biolegend, USA) according to manufacturer’s specifications.

**Statistical analysis:**

**In Table 1:** gender was analyzed by chi-square while prior IDU, previous use of ddI/d4T, present ARV based on IP, present ARV based on NNRTI, present ARV based on INSTI, previous IFN/PEGIFN and HCV genotype, by Fisher’s exact test. Age distribution was analyzed by Kruskal-Wallis-test, and CD4/CD8 absolute numbers and APRI score by means of one–way ANOVA. HCV viral load, ALT, AST, albumin and bilirubin levels, as well as prothrombin time were compared by means of Mann-Whitney test, while time of known HCV, HIV infection and antiretroviral treatment, liver stiffness, γ-GT, and platelet levels were analyzed using the Student´s t- test. **In Table 2**, cell frequencies were compared using Kruskal Wallis test. When multiple comparisons were perform, p values were corrected using statistical hypothesis testing (Dunn’s for non-parametrical and Tukey for parametrical tests). Wilcoxon matched-paired test was applied in order to analyze paired data. Correlations between variables were quantified with Sperman´s Rank correlation coefficient.

**Table S1.** Fluorochrome-conjugated antibody panels.

| **Antibody** | **Fluorochrome** | **Clone** | **Supplier** |
| --- | --- | --- | --- |
| **NK functional assays** |  |  |  |
| Anti-CD3 | PE-CY7 | SK7 | Biolegend |
| Anti-CD56 | PE-CY5 | 679.1Mc7 | Beckman Coulter |
| *CD107a assay* |  |  |  |
| Anti-CD107a | FITC | H4A3 | Biolegend |
| *IFN-γ and TNF-α secretion* |  |  |  |
| Anti-IFN-γ | APC | 4S.B3 | Biolegend |
| Anti-TNF-α | FITC | mAb11 | Biolegend |
| **NK immunophenotyping** |  |  |  |
| Anti-CD3 | BV605 | SK7 | BD Biosciences |
| Anti-NKp46 | PE-CY7 | 9E2 | Biolegend |
| Anti-NKG2D | APC | 1D11 | Biolegend |
| Anti-CD25 | PE | BC96 | Biolegend |
| Anti-CD69 | FITC | FN50 | Biolegend |
| Anti-PD-1 | BV421 | EH12.1 | BD Biosciences |
| **CD4+ T cell activation** |  |  |  |
| Anti-CD3 | PE-CY7 | SK7 | Biolegend |
| Anti-CD4 | PerCP | OKT4 | Biolegend |
| Anti-CD25 | PE | BC96 | Biolegend |
| Anti-CD69 | FITC | FN50 | Biolegend |
| Anti-CD38 | APC | HB7 | BD Biosciences |

**Table S2:** Differences in NK and CD4+ T-cell phenotypic and functional markers according gender.

| **Indices** | **Experimental group** | **Female** | **Male** | **p value** |
| --- | --- | --- | --- | --- |
| NK cell degranulation  (fold change %CD107a+ NK cells) | Healthy donors | 3.33 ± 0.56 | 3.59 ± 1.33 | 0.52 |
|  | F0/F1 | 3.52 ± 1.02 | 3.96 ± 0.79 | 0.18 |
|  | F4 | 2.73 ± 1.06 | 2.16 ± 2. 40 | 0.82 |
| IFN-γ (%IFN-γ+ NK cells) | Healthy donors | 14.67 ± 4.51 | 12.76 ± 2.98 | 0.62 |
|  | F0/F1 | 9.60 ± 2.91 | 14.25 ± 5.61 | 0.17 |
|  | F4 | 7.91 ± 0.7 | 7.47 ± 2.44 | 0.71 |
| TNF-α (%TNF-α+ NK cells) | Healthy donors | 20.4 ± 9.65 | 23.97 ± 3.10 | >0.90 |
|  | F0/F1 | 14.98 ± 5.93 | 14.38 ± 8.32 | 0.87 |
|  | F4 | 9.47 | 9.63 ± 4.78 | - |
| PD-1 (% of positive NK cells) | F0/F1 | 16.73 ± 6.92 | 19.55 ± 9.14 | 0.39 |
|  | F4 | 35.1 | 29.04 ± 8.53 | - |
| IL-2 (ng/ml) **^1^** | F0/F1 | 10.52 ± 3.09 | 19.60 ± 8.59 | 0.19 |
|  | F4 | 11.67 | 14.28 ± 7.11 | - |
| CD69+/CD4^+^ T-cells (%) **^1^** | F0/F1 | 78.09 ± 13.47 | 82.29 ± 5.62 | 0.84 |
|  | F4 | 88.35 | 82.53 ± 7.62 | - |
| CD25+/CD4^+^ T-cells (%) **^1^** | F0/F1 | 76.05 ± 10.70 | 77.42 ± 5.51 | >0.9 |
|  | F4 | 84.75 | 79.8 ± 7.24 | - |
| CD38+/CD4^+^ T-cells (%) **^1^** | F0/F1 | 65.9 ± 7.99 | 57.02 ± 17.89 | 0.69 |
|  | F4 | 60.45 | 56.18 ± 14.6 | - |

Indices are expressed as media ± SD. Comparisons between genders were perform by using Mann-Whitney test. In TNF-α, PD-1, IL-2, CD69, CD25 and CD38 indices, only one female with advanced fibrosis was analyzed, and so no statistically analysis was possible. **1**- Obtained at CD4^+^ T-cell to CD3/CD28 ratio of 1:1.


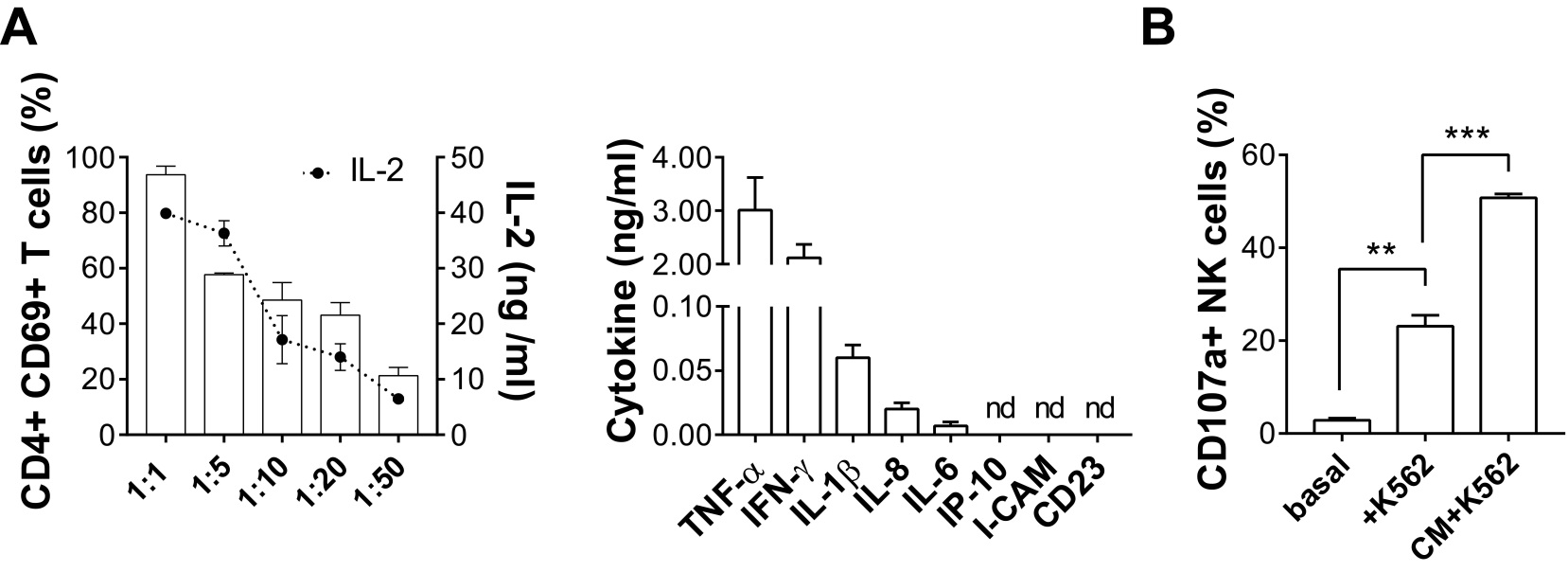


**Figure S1. Lymphocytes isolated from a selected healthy volunteer.** NK and CD4^+^ T-cells were purified from PBMC of a 42- year- old woman, HIV/HCV/HBV negative, with no history of alcohol consumption, tabaquism or ilicit drug use, thyroid or celiac disease, and no other clinically relevant conditions. % NK cells: 8.62, CD4+ T-cell count: 754, % CD4^+^ T-cells: 38. **(A)** CD4+ T-cells were stimulated with anti-CD3/CD28 beads in different bead-to-cell ratios (1:1, 48 h; others 24 h), and percentages of CD69+/CD4+ T cells were monitored. IL-2 levels were also measured in corresponding culture supernatants. Right: Additional cytokines were quantified in CD4+ T-cell CM (1:1, 48h). **(B)** CD107a externalization in CM-pre-stimulated PBMC, co-cultured with K562 cells (CM+K562). As control, cRPMI-pre-stimulated PBMC were either exposed to K562 cells (+K562) or cRPMI (basal). Determinations were performed in duplicate. Data is presented as mean ± SD. Statistical comparisons were performed using Wilcoxon matched paired test. nd: not detected.


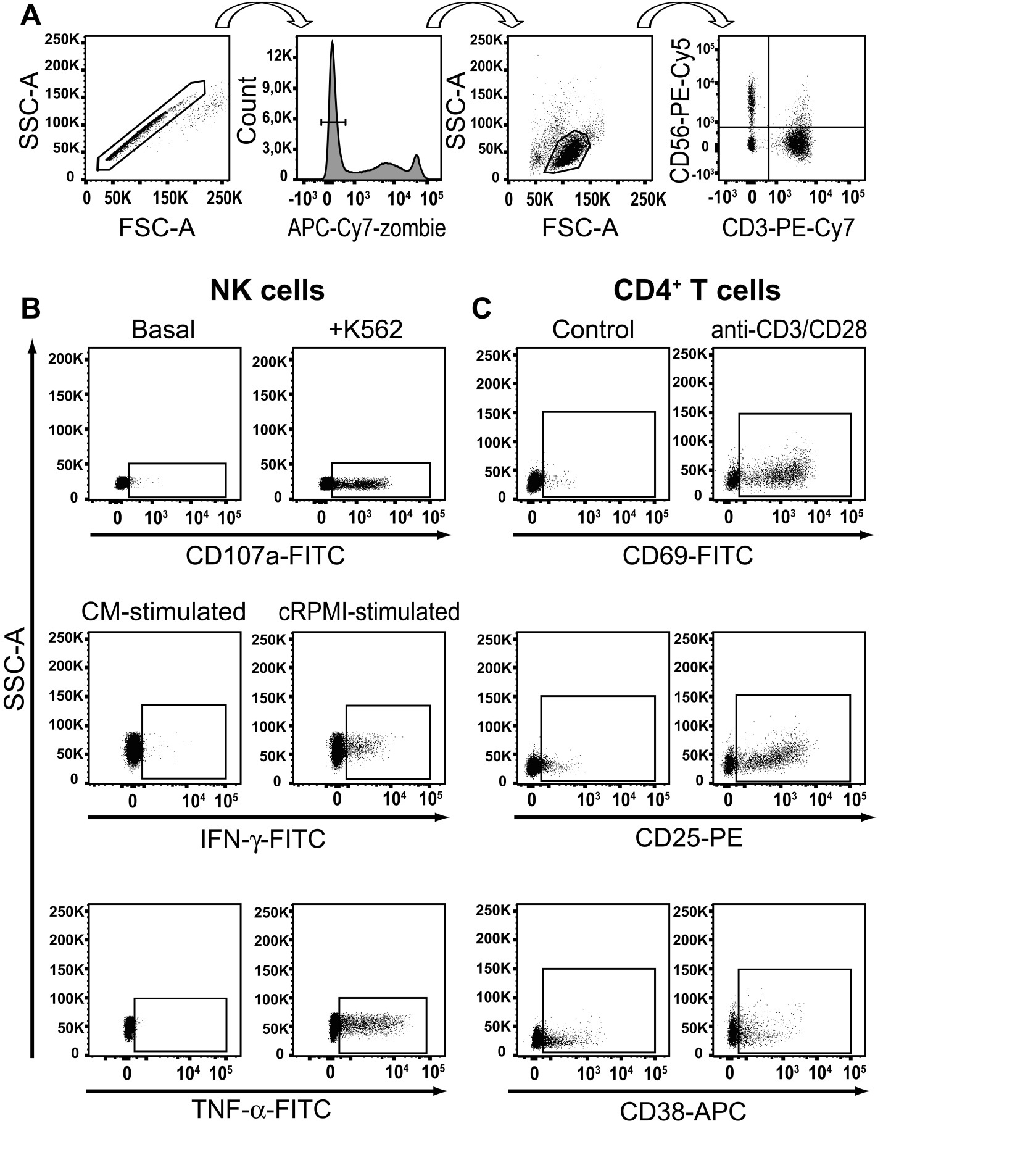


**Figure S2.** [**Gating strategy, and representative dot plots for flow cytometry analysis.**](https://figshare.com/articles/_Gating_strategy_and_representative_dot_plots_for_flow_cytometry_analysis_/903493) **(A)** NK cell subset was defined as CD3-/CD56+ viable lymphocytes. **(B)** Upon incubation with cRPMI (basal) or K562 cells (+K562), CD107a+ NK cells were determined (Top). Intracellular IFN-γ and TNF-α expression was measured in cRPMI or CM-pre-stimulated NK cells, co-cultured with K562 cells (Bottom). **(C)** CD69+, CD25+ or CD38+ cells were determined in vehicle (control) or anti-CD3/CD28-stimulated CD4^+^ T-cells. At least one thousand events were acquired for both NK and CD4^+^ T-cell gates.


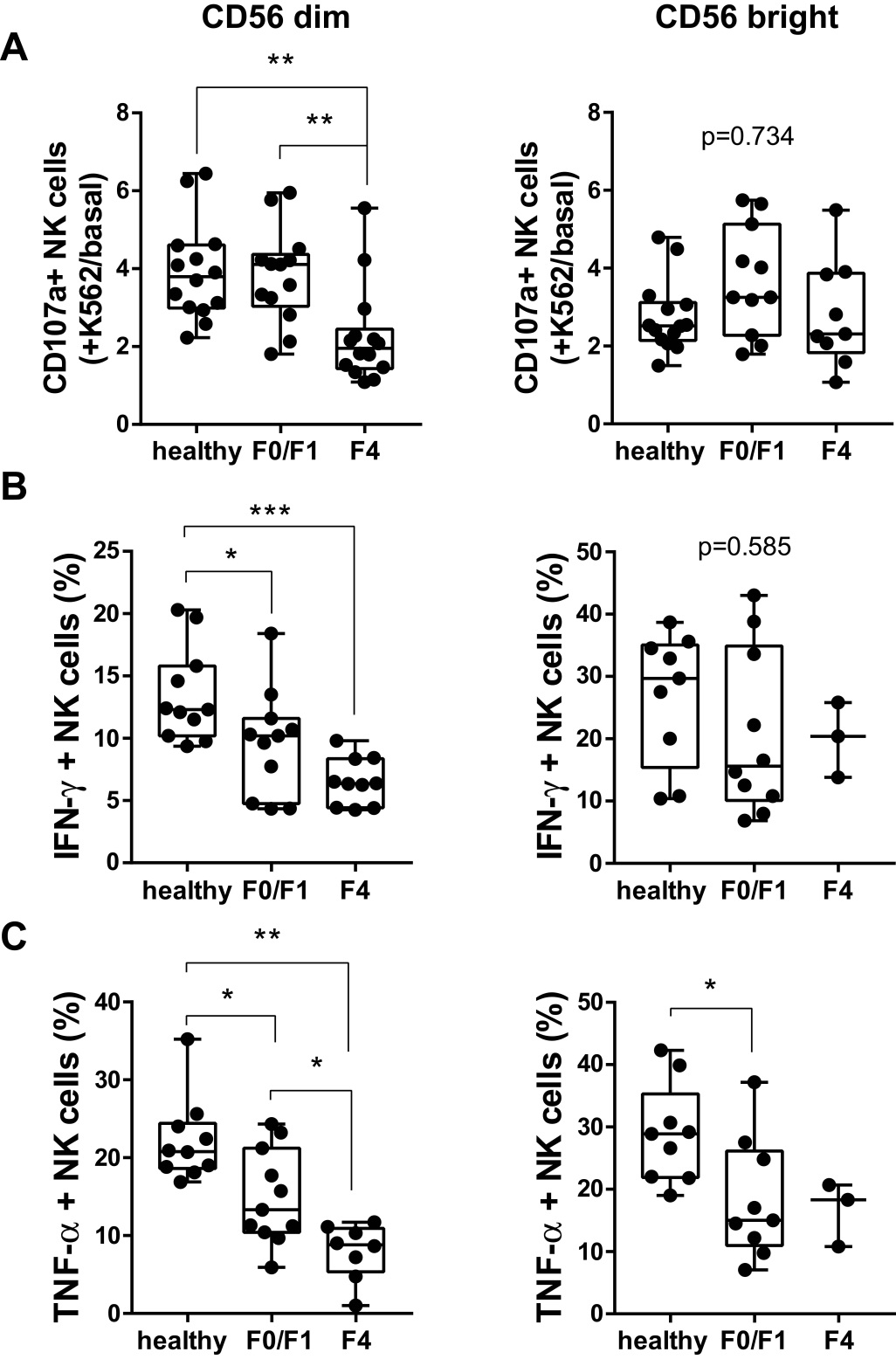


**Figure S3. Evaluation of NK cell effector functions in CD56 dim and bright populations.** **(A)** PBMCs from healthy and HIV/HCV-coinfected individuals with METAVIR F0/F1 or F4 scores were incubated with cRPMI (basal) or K562 cells (+K562). Fold change induction in CD107a expression (+K562/basal) was evaluated in CD56^dim^ and CD56^bright^ cell subsets. **(B, C):** For cytokine expression, PBMCs cells were pre-treated with conditioned medium from CD4^+^ T-lymphocytes, and subsequently exposed to K562 cells. Frequencies of IFN-γ (B) and TNF-α-positive cells (C) were determined in CD56^dim^ and CD56^bright^ cell subsets. Statistical analysis was performed using Kruskal-Wallis followed by Dunn´s multiple-comparison.

**References:**

1. Warren HS, Rana PM. An economical adaptation of the RosetteSep procedure for NK cell enrichment from whole blood, and its use with liquid nitrogen stored peripheral blood mononuclear cells. Journal of immunological methods. 2003;280(1-2):135-8.
